# Supplementary material for: The complete chloroplast genomes of seventeen Aegilops tauschii: genome comparative analysis and phylogenetic inference
Source: PeerJ. 2020 Mar 4;8:e8678. doi: 10.7717/peerj.8678 (PMC7060751; doi:10.7717/peerj.8678)
Supplement: Table S1 [file peerj-08-8678-s002.docx]

**Table S1 List of genes present in the *Aegilops tauschii* chloroplast genome**

| Category | Gene Group | Gene Name | |
| --- | --- | --- | --- |
| Self-replication | Ribosomal RNA genes | rrn4.5S^a^ rrn5S^a^ rrn16S^a^  rrn23S^a^ | |
|  | Transfer RNA genes | trnC-GCA trnD-GUC trnE-UUC trnF-GAA trnG-GCC | |
|  |  | trnH-GUG^a^ trnI-CAU^a^ trnL-UAG trnL-CAA^a^ trnM-CAU  trnN-GUU^a^ trnA-UGC^a^ | |
|  |  | trnP-UGG trnQ-UUG trnR-UCU trnR-ACG^a^ trnS-GCU trnS-GGA |  |
|  |  | trnS-UGA trnT-GGU trnT-UGU trnV-GAC^a^ trnW-CCA |  |
|  |  | trnY-GUA trnfM-CAU^1^ trnfM-CAU^2^  tRNAK |  |
|  | Small subunit of ribosome | rps2 rps3 rps4 rps7^a^ rps8 rps11 rps12^b^ rps14 rps15^a^ |  |
|  |  | rps16 rps18 rps19^a^ |  |
|  | Large subunit of ribosome | rpl2^a,b^ rpl14 rpl16 rpl20 rpl22 rpl23^a^ rpl32 rpl33 rpl36 |  |
|  | DNA-dependent RNA polymerase | rpoA rpoB rpoC1 rpoC2 |  |
|  | Translational initiation factor | infA |  |
| photosynthesis | Subunits of photosystem I | psaA psaB psaC psaI psaJ ycf3^c^ ycf4 |  |
|  | Subunits of photosystem II | psbA psbB psbC psbD psbE psbF psbH psbI |  |
|  |  | psbJ psbK psbL psbM psbN psbT psbZ |  |
|  | NADH oxidoreductase | ndhA^b^ ndhB^a,b^ ndhC ndhD ndhE ndhF ndhG ndhH ndhI ndhJ ndhK |  |
|  | Subunits of cytochrome | petA petB petD petG petN petL |  |
|  | Subunits of ATP synthase | atpA atpB atpE atpF^b^ atpH atpI |  |
|  | Large subunit of Rubisco | rbcL |  |
| Other genes | Maturase | matk |  |
|  | Envelope membrane protein | cemA |  |
|  | C-type cytochrome synthesis gene | ccsA |  |
|  | Protease | clpP |  |

a Two gene copies in IRs; b gene containing a single intron; c gene containing two introns;

1，2 represent for two genes in different CDS
