# Supplementary material for: The complete chloroplast genomes of seventeen Aegilops tauschii: genome comparative analysis and phylogenetic inference
Source: PeerJ. 2020 Mar 4;8:e8678. doi: 10.7717/peerj.8678 (PMC7060751; doi:10.7717/peerj.8678)
Supplement: Table S2 [file peerj-08-8678-s003.docx]

| GO terms | Function class | GO-ID | Number of genes | Genes |
| --- | --- | --- | --- | --- |
| molecular_function | electron carrier activity | GO:0009055 | 12 | psaC(GO:0009055);psbC(GO:0045156);petD(GO:0045158,GO:0045156);psbE(GO:0009055);psbD(GO:0045156);petB(GO:0045158);petG(GO:0045158);petA(GO:0009055);psaA(GO:0009055);psaB(GO:0009055);psbA(GO:0045156);psbF(GO:0009055) |
| molecular_function | structural molecule activity | GO:0005198 | 21 | rps18(GO:0003735);rps2(GO:0003735);rpl33(GO:0003735);rpl20(GO:0003735);rpl23(GO:0003735);rps19(GO:0003735);rps16(GO:0003735);rpl32(GO:0003735);rpl14(GO:0003735);rps14(GO:0003735);rps7(GO:0003735);rpl36(GO:0003735);rpl22(GO:0003735);rps8(GO:0003735);rpl16(GO:0003735);rps11(GO:0003735);rps15(GO:0003735);rps12(GO:0003735);rpl2(GO:0003735);rps4(GO:0003735);rps3(GO:0003735) |
| molecular_function | binding | GO:0005488 | 50 | rps18(GO:0019843);ndhC(GO:0048038);psbL(GO:0046872,GO:0020037);atpH(GO:0008289);ndhI(GO:0005506,GO:0051539,GO:0048038);rpl20(GO:0019843);ndhA(GO:0048038,GO:0051287);rpl23(GO:0000166,GO:0019843);rps19(GO:0019843);ndhG(GO:0048038);ndhK(GO:0005506,GO:0051539,GO:0048038);matK(GO:0003723);ndhF(GO:0048038);rpl14(GO:0019843);psbF(GO:0020037,GO:0005506);psaC(GO:0046872,GO:0051539);rpl22(GO:0019843);rps7(GO:0019843);psbE(GO:0020037,GO:0005506);rps8(GO:0019843);petB(GO:0020037,GO:0005506);rpl16(GO:0019843);ndhB(GO:0048038);petG(GO:0005507);petA(GO:0020037,GO:0005506);psbH(GO:0042301);rpl2(GO:0003723);ndhH(GO:0048038,GO:0051287);rpoC2(GO:0003677);rps3(GO:0019843);ndhJ(GO:0048038);rbcL(GO:0000287);psbB(GO:0016168);rpoA(GO:0003677,GO:0046983);atpB(GO:0005524);psbD(GO:0005506);ndhE(GO:0048038);psaA(GO:0000287,GO:0051539,GO:0016168);infA(GO:0003743);rpoB(GO:0003677,GO:0032549);rps14(GO:0019843);psbC(GO:0016168);ndhD(GO:0048038);atpA(GO:0005524);rpoC1(GO:0003677);atpE(GO:0005524);rps11(GO:0003677,GO:0019843,GO:0046983);psaB(GO:0000287,GO:0051539,GO:0016168);psbA(GO:0005506);rps4(GO:0019843) |
| molecular_function | transporter activity | GO:0005215 | 9 | cemA(GO:0015078);atpI(GO:0046933);atpH(GO:0015078);atpB(GO:0046933);atpA(GO:0046933,GO:0046961);atpF(GO:0046933);atpE(GO:0046933,GO:0046961);petN(GO:0045158);petL(GO:0045158); |
| molecular_function | catalytic activity | GO:0003824 | 32 | ndhC(GO:0008137);ndhI(GO:0008137);ndhA(GO:0016655);rps19(GO:0016740);ndhG(GO:0008137);ndhK(GO:0008137);ndhF(GO:0008137);atpF(GO:0046933);psaC(GO:0016491);petB(GO:0016491);ndhB(GO:0008137);ndhH(GO:0016655);rpl2(GO:0016740);clpP(GO:0004252);rpoC2(GO:0003899);rbcL(GO:0004497,GO:0016984);ndhJ(GO:0008137);atpI(GO:0046933);rpoA(GO:0003899);atpB(GO:0046933);psbD(GO:0016491);ndhE(GO:0050136);psaA(GO:0016491);rpoB(GO:0003899);petD(GO:0016491);ndhD(GO:0008137);rpoC1(GO:0003899);atpA(GO:0046933,GO:0046961);atpE(GO:0046933,GO:0046961);rps11(GO:0003899);psaB(GO:0016491);psbA(GO:0016491) |
| cellular_component | organelle | GO:0043226 | 74 | atpH(GO:0009535,GO:0016023);ndhI(GO:0009535);psbK(GO:0009507);rpl20(GO:0005840,GO:0009507);matK(GO:0009507);ndhF(GO:0009507);atpF(GO:0009535);psbF(GO:0009535,GO:0016023);rps7(GO:0005763,GO:0022627,GO:0009507);psbE(GO:0009535);petB(GO:0009535);rpl16(GO:0005840,GO:0009507);petG(GO:0009535,GO:0016023,GO:0009570);petA(GO:0009535);rps12(GO:0015935,GO:0009507);ndhH(GO:0009535);rpl2(GO:0015934,GO:0005739,GO:0015935,GO:0009507);clpP(GO:0009570);rps3(GO:0005739,GO:0015935,GO:0009507);rps2(GO:0005739,GO:0015935,GO:0009507);atpB(GO:0009535);ndhE(GO:0009535,GO:0016023);psaA(GO:0009535);psbT(GO:0009535);psbM(GO:0009535,GO:0005739);infA(GO:0005840,GO:0009507);rpoB(GO:0005730,GO:0009507);rpl36(GO:0005840,GO:0009507);psbC(GO:0009535);ndhD(GO:0009535);rpoC1(GO:0005730,GO:0009507);atpE(GO:0009535);psbN(GO:0009535,GO:0016023);rps11(GO:0005840,GO:0005730,GO:0009507);rps4(GO:0005739,GO:0015935,GO:0009507);psaI(GO:0009535);ndhC(GO:0009535);psaJ(GO:0009535,GO:0016023);rps18(GO:0005840,GO:0009507);psbL(GO:0009535);rpl33(GO:0005840,GO:0009507);ndhA(GO:0009535);rpl23(GO:0005840,GO:0009507);rps19(GO:0015934,GO:0015935,GO:0009507);ndhG(GO:0009535);rpl32(GO:0015934,GO:0009507);ndhK(GO:0009535);rpl14(GO:0015934,GO:0009507);psaC(GO:0009535);psbJ(GO:0009535,GO:0016023);rpl22(GO:0015934,GO:0009507);rps8(GO:0005840,GO:0009507);ndhB(GO:0009535);psbH(GO:0009535);rpoC2(GO:0005730,GO:0009507);rbcL(GO:0009573);ndhJ(GO:0009535);atpI(GO:0009535);psbB(GO:0009535);rpoA(GO:0005730,GO:0009507);ycf3(GO:0009535);psbD(GO:0009535);cemA(GO:0009706);rps16(GO:0005840,GO:0009507);ccsA(GO:0009535);psbZ(GO:0009535);rps14(GO:0005840,GO:0005739,GO:0009507);petD(GO:0009535);atpA(GO:0009535,GO:0005743);psaB(GO:0009535);rps15(GO:0005840,GO:0009507);psbI(GO:0009535);psbA(GO:0009535);ycf4(GO:0009535) |
| cellular_component | cell part | GO:0044464 | 74 | atpH(GO:0009535,GO:0045263,GO:0016023);ndhI(GO:0009535);psbK(GO:0009539,GO:0009507);rpl20(GO:0005840,GO:0009507);matK(GO:0009507);ndhF(GO:0009507);atpF(GO:0009535,GO:0045263,GO:0005886);psbF(GO:0009539,GO:0009535,GO:0016023);rps7(GO:0030686,GO:0032040,GO:0005763,GO:0022627,GO:0009507);psbE(GO:0009539,GO:0009535);petB(GO:0009535);rpl16(GO:0005840,GO:0009507);petG(GO:0031977,GO:0009535,GO:0009512,GO:0016023,GO:0009570);petA(GO:0009535,GO:0031361);rps12(GO:0015935,GO:0009507);ndhH(GO:0009535);rpl2(GO:0015934,GO:0005739,GO:0015935,GO:0009507);clpP(GO:0009570);rps3(GO:0005739,GO:0015935,GO:0009507);rps2(GO:0005739,GO:0015935,GO:0009507);atpB(GO:0045261,GO:0009535,GO:0045263,GO:0005886);ndhE(GO:0009535,GO:0016023,GO:0005886);psaA(GO:0009535,GO:0009522);psbT(GO:0009539,GO:0009535);psbM(GO:0009535,GO:0009523,GO:0005739);infA(GO:0005840,GO:0009507);rpoB(GO:0005730,GO:0009507);rpl36(GO:0005840,GO:0009507);psbC(GO:0009535,GO:0009523,GO:0030076);ndhD(GO:0009535);rpoC1(GO:0005730,GO:0009507);atpE(GO:0045261,GO:0009535,GO:0005886);psbN(GO:0009539,GO:0009535,GO:0016023);rps11(GO:0005840,GO:0005730,GO:0009507);rps4(GO:0005739,GO:0015935,GO:0009507);psaI(GO:0009535,GO:0009522);ndhC(GO:0009535);psaJ(GO:0009535,GO:0016023,GO:0009522);rps18(GO:0005840,GO:0009507);psbL(GO:0009539,GO:0009535);rpl33(GO:0005840,GO:0009507);ndhA(GO:0009535);rpl23(GO:0005840,GO:0009507);rps19(GO:0015934,GO:0015935,GO:0009507);ndhG(GO:0009535);rpl32(GO:0015934,GO:0009507);ndhK(GO:0009535,GO:0005886);rpl14(GO:0015934,GO:0009507);psaC(GO:0009535,GO:0009522);psbJ(GO:0009539,GO:0009535,GO:0016023);rpl22(GO:0015934,GO:0009507);rps8(GO:0005840,GO:0009507);ndhB(GO:0009535,GO:0005886);psbH(GO:0009535,GO:0009523);rpoC2(GO:0005730,GO:0009507);rbcL(GO:0009573);ndhJ(GO:0009535);atpI(GO:0009535,GO:0045263,GO:0005886);psbB(GO:0009535,GO:0009523);rpoA(GO:0005730,GO:0009507);ycf3(GO:0009535);psbD(GO:0009535,GO:0009523);cemA(GO:0009706);rps16(GO:0005840,GO:0009507);ccsA(GO:0009535);psbZ(GO:0009539,GO:0009535);rps14(GO:0005840,GO:0005739,GO:0009507);petD(GO:0009535);atpA(GO:0045261,GO:0009535,GO:0045263,GO:0005743,GO:0005886);psaB(GO:0009535,GO:0009522);rps15(GO:0005840,GO:0009507);psbI(GO:0009539,GO:0009535);psbA(GO:0009535,GO:0009523);ycf4(GO:0009535,GO:0009522) |
| cellular_component | membrane-enclosed lumen | GO:0031974 | 6 | rps11(GO:0005730);rpoB(GO:0005730);rpoA(GO:0005730);rps7(GO:0005763);rpoC1(GO:0005730);rpoC2(GO:0005730) |
| cellular_component | membrane part | GO:0044425 | 40 | psaJ(GO:0016021,GO:0009522);ndhC(GO:0016021);psbL(GO:0009539,GO:0016021);atpH(GO:0045263,GO:0016021);psbK(GO:0009539);ndhA(GO:0016021);ndhG(GO:0016021);ndhF(GO:0016021);atpF(GO:0045263,GO:0016021);psbF(GO:0009539,GO:0016021);psaC(GO:0009522);psbJ(GO:0009539,GO:0016021);psbE(GO:0009539,GO:0016021);petB(GO:0016021);ndhB(GO:0016021);petG(GO:0016021);petA(GO:0031361);psbH(GO:0009523,GO:0016021);atpI(GO:0045263,GO:0016021);psbB(GO:0009523,GO:0016021);atpB(GO:0045261,GO:0045263,GO:0016021);psbD(GO:0009523,GO:0016021);ndhE(GO:0016021);cemA(GO:0016021);psaA(GO:0016021,GO:0009522);ccsA(GO:0016021);psbT(GO:0009539,GO:0016021);psbZ(GO:0009539,GO:0016021);psbM(GO:0009523,GO:0016021);psbC(GO:0009523,GO:0016021);petD(GO:0016021);ndhD(GO:0016021);atpA(GO:0045261,GO:0045263);atpE(GO:0045261);psbN(GO:0009539,GO:0016021);psaB(GO:0016021,GO:0009522);psbI(GO:0009539,GO:0016021);psbA(GO:0009523,GO:0016021);ycf4(GO:0016021,GO:0009522);psaI(GO:0016021,GO:0009522) |
| cellular_component | cell | GO:0005623 | 74 | atpH(GO:0009535,GO:0045263,GO:0016023);ndhI(GO:0009535);psbK(GO:0009539,GO:0009507);rpl20(GO:0005840,GO:0009507);matK(GO:0009507);ndhF(GO:0009507);atpF(GO:0009535,GO:0045263,GO:0005886);psbF(GO:0009539,GO:0009535,GO:0016023);rps7(GO:0030686,GO:0032040,GO:0005763,GO:0022627,GO:0009507);psbE(GO:0009539,GO:0009535);petB(GO:0009535);rpl16(GO:0005840,GO:0009507);petG(GO:0031977,GO:0009535,GO:0009512,GO:0016023,GO:0009570);petA(GO:0009535,GO:0031361);rps12(GO:0015935,GO:0009507);ndhH(GO:0009535);rpl2(GO:0015934,GO:0005739,GO:0015935,GO:0009507);clpP(GO:0009570);rps3(GO:0005739,GO:0015935,GO:0009507);rps2(GO:0005739,GO:0015935,GO:0009507);atpB(GO:0045261,GO:0009535,GO:0045263,GO:0005886);ndhE(GO:0009535,GO:0016023,GO:0005886);psaA(GO:0009535,GO:0009522);psbT(GO:0009539,GO:0009535);psbM(GO:0009535,GO:0009523,GO:0005739);infA(GO:0005840,GO:0009507);rpoB(GO:0005730,GO:0009507);rpl36(GO:0005840,GO:0009507);psbC(GO:0009535,GO:0009523,GO:0030076);ndhD(GO:0009535);rpoC1(GO:0005730,GO:0009507);atpE(GO:0045261,GO:0009535,GO:0005886);psbN(GO:0009539,GO:0009535,GO:0016023);rps11(GO:0005840,GO:0005730,GO:0009507);rps4(GO:0005739,GO:0015935,GO:0009507);psaI(GO:0009535,GO:0009522);ndhC(GO:0009535);psaJ(GO:0009535,GO:0016023,GO:0009522);rps18(GO:0005840,GO:0009507);psbL(GO:0009539,GO:0009535);rpl33(GO:0005840,GO:0009507);ndhA(GO:0009535);rpl23(GO:0005840,GO:0009507);rps19(GO:0015934,GO:0015935,GO:0009507);ndhG(GO:0009535);rpl32(GO:0015934,GO:0009507);ndhK(GO:0009535,GO:0005886);rpl14(GO:0015934,GO:0009507);psaC(GO:0009535,GO:0009522);psbJ(GO:0009539,GO:0009535,GO:0016023);rpl22(GO:0015934,GO:0009507);rps8(GO:0005840,GO:0009507);ndhB(GO:0009535,GO:0005886);psbH(GO:0009535,GO:0009523);rpoC2(GO:0005730,GO:0009507);rbcL(GO:0009573);ndhJ(GO:0009535);atpI(GO:0009535,GO:0045263,GO:0005886);psbB(GO:0009535,GO:0009523);rpoA(GO:0005730,GO:0009507);ycf3(GO:0009535);psbD(GO:0009535,GO:0009523);cemA(GO:0009706);rps16(GO:0005840,GO:0009507);ccsA(GO:0009535);psbZ(GO:0009539,GO:0009535);rps14(GO:0005840,GO:0005739,GO:0009507);petD(GO:0009535);atpA(GO:0045261,GO:0009535,GO:0045263,GO:0005743,GO:0005886);psaB(GO:0009535,GO:0009522);rps15(GO:0005840,GO:0009507);psbI(GO:0009539,GO:0009535);psbA(GO:0009535,GO:0009523);ycf4(GO:0009535,GO:0009522) |
| cellular_component | macromolecular complex | GO:0032991 | 51 | rps18(GO:0005840);psaJ(GO:0009522);rpl33(GO:0005840);psbL(GO:0009539);atpH(GO:0045263);psbK(GO:0009539);rpl20(GO:0005840);rpl23(GO:0005840);rps19(GO:0015934,GO:0015935);rpl32(GO:0015934);atpF(GO:0045263);rpl14(GO:0015934);psbF(GO:0009539);psaC(GO:0009522);psbJ(GO:0009539);rpl22(GO:0015934);rps7(GO:0030686,GO:0032040,GO:0005763,GO:0022627);psbE(GO:0009539);rps8(GO:0005840);rpl16(GO:0005840);petG(GO:0009512);psbH(GO:0009523);rps12(GO:0015935);rpl2(GO:0015934,GO:0015935);rps3(GO:0015935);rbcL(GO:0009573);rps2(GO:0015935);psbB(GO:0009523);atpI(GO:0045263);atpB(GO:0045261,GO:0045263);psbD(GO:0009523);rps16(GO:0005840);psaA(GO:0009522);psbZ(GO:0009539);psbT(GO:0009539);psbM(GO:0009523);infA(GO:0005840);rps14(GO:0005840);rpl36(GO:0005840);psbC(GO:0009523,GO:0030076);atpA(GO:0045261,GO:0045263);atpE(GO:0045261);psbN(GO:0009539);rps11(GO:0005840);psaB(GO:0009522);rps15(GO:0005840);psbI(GO:0009539);psbA(GO:0009523);rps4(GO:0015935);ycf4(GO:0009522);psaI(GO:0009522) |
| cellular_component | membrane | GO:0016020 | 45 | psaJ(GO:0009535,GO:0016021,GO:0009522);ndhC(GO:0009535,GO:0016021);psbL(GO:0009539,GO:0009535,GO:0016021);atpH(GO:0009535,GO:0045263,GO:0016021);ndhI(GO:0009535);psbK(GO:0009539);ndhA(GO:0009535,GO:0016021);ndhG(GO:0009535,GO:0016021);ndhK(GO:0009535,GO:0005886);ndhF(GO:0016021);atpF(GO:0009535,GO:0045263,GO:0016021,GO:0005886);psbF(GO:0009539,GO:0009535,GO:0016021);psaC(GO:0009535,GO:0009522);psbJ(GO:0009539,GO:0009535,GO:0016021);psbE(GO:0009539,GO:0009535,GO:0016021);petB(GO:0009535,GO:0016021);ndhB(GO:0009535,GO:0016021,GO:0005886);petG(GO:0009535,GO:0016021);petA(GO:0009535,GO:0031361);psbH(GO:0009535,GO:0009523,GO:0016021);ndhH(GO:0009535);ndhJ(GO:0009535);psbB(GO:0009535,GO:0009523,GO:0016021);atpI(GO:0009535,GO:0045263,GO:0016021,GO:0005886);ycf3(GO:0009535);atpB(GO:0045261,GO:0009535,GO:0045263,GO:0016021,GO:0005886);psbD(GO:0009535,GO:0009523,GO:0016021);ndhE(GO:0009535,GO:0016021,GO:0005886);cemA(GO:0016021,GO:0009706);psaA(GO:0009535,GO:0016021,GO:0009522);ccsA(GO:0009535,GO:0016021);psbZ(GO:0009539,GO:0009535,GO:0016021);psbT(GO:0009539,GO:0009535,GO:0016021);psbM(GO:0009535,GO:0009523,GO:0016021);psbC(GO:0009535,GO:0009523,GO:0016021);petD(GO:0009535,GO:0016021);ndhD(GO:0009535,GO:0016021);atpA(GO:0045261,GO:0009535,GO:0045263,GO:0005743,GO:0005886);atpE(GO:0045261,GO:0009535,GO:0005886);psbN(GO:0009539,GO:0009535,GO:0016021);psaB(GO:0009535,GO:0016021,GO:0009522);psbI(GO:0009539,GO:0009535,GO:0016021);psbA(GO:0009535,GO:0009523,GO:0016021);ycf4(GO:0009535,GO:0016021,GO:0009522);psaI(GO:0009535,GO:0016021,GO:0009522) |
| cellular_component | organelle part | GO:0044422 | 60 | psaJ(GO:0009535);ndhC(GO:0009535);psbL(GO:0009535);atpH(GO:0009535);ndhI(GO:0009535);ndhA(GO:0009535);rps19(GO:0015934,GO:0015935);ndhG(GO:0009535);ndhK(GO:0009535);rpl32(GO:0015934);atpF(GO:0009535);rpl14(GO:0015934);psbF(GO:0009535);psaC(GO:0009535);psbJ(GO:0009535);rpl22(GO:0015934);rps7(GO:0005763,GO:0022627);psbE(GO:0009535);petB(GO:0009535);ndhB(GO:0009535);petG(GO:0009535,GO:0009570);petA(GO:0009535);psbH(GO:0009535);rps12(GO:0015935);clpP(GO:0009570);rpl2(GO:0015934,GO:0015935);ndhH(GO:0009535);rpoC2(GO:0005730);rps3(GO:0015935);ndhJ(GO:0009535);rbcL(GO:0009573);rps2(GO:0015935);psbB(GO:0009535);atpI(GO:0009535);rpoA(GO:0005730);ycf3(GO:0009535);atpB(GO:0009535);psbD(GO:0009535);ndhE(GO:0009535);cemA(GO:0009706);psaA(GO:0009535);ccsA(GO:0009535);psbZ(GO:0009535);psbT(GO:0009535);psbM(GO:0009535);rpoB(GO:0005730);psbC(GO:0009535);petD(GO:0009535);ndhD(GO:0009535);atpA(GO:0009535,GO:0005743);rpoC1(GO:0005730);atpE(GO:0009535);psbN(GO:0009535);rps11(GO:0005730);psaB(GO:0009535);psbI(GO:0009535);psbA(GO:0009535);rps4(GO:0015935);ycf4(GO:0009535);psaI(GO:0009535) |
| biological_process | regulation of biological process | GO:0050789 | 4 | infA(GO:0006446);petG(GO:0017148);psbH(GO:0050821);psbZ(GO:0042549) |
| biological_process | establishment of localization | GO:0051234 | 17 | ndhJ(GO:0015992,GO:0006814);ndhC(GO:0006814,GO:0015992);atpI(GO:0015986);atpH(GO:0015986,GO:0015991);ndhI(GO:0006814,GO:0015992);atpB(GO:0015986,GO:0015991);ndhG(GO:0015992,GO:0006814);ndhE(GO:0006810);cemA(GO:1902600);ndhK(GO:0006814,GO:0015992);ndhF(GO:0015992,GO:0006814);atpF(GO:0015986);ndhD(GO:0015992,GO:0006814);atpA(GO:0015986,GO:0015991);atpE(GO:0015986,GO:0015991);ndhB(GO:0006814,GO:0015992);ndhH(GO:0006810) |
| biological_process | cellular component organization or biogenesis | GO:0071840 | 23 | rps18(GO:0042254);rps2(GO:0042254);rpl33(GO:0042254);rpl20(GO:0042254);rpl23(GO:0042254);rps19(GO:0042254);rps16(GO:0042254);rpl32(GO:0042254);ccsA(GO:0008535);rpl14(GO:0042254);rps14(GO:0042254);rpl22(GO:0042254);rps7(GO:0042274,GO:0006364);rpl36(GO:0042254);rps8(GO:0042254);rpl16(GO:0042254);petG(GO:0017004);rps11(GO:0042254);rps15(GO:0042254);rps12(GO:0042254);rpl2(GO:0042254);rps4(GO:0042254);rps3(GO:0042254) |
| biological_process | cellular process | GO:0009987 | 72 | atpH(GO:0015986,GO:0015991);ndhI(GO:0006120,GO:0019684,GO:0006744);psbK(GO:0015979);rpl20(GO:0006412);matK(GO:0006397,GO:0008380,GO:0008033);ndhF(GO:0006120,GO:0006744);atpF(GO:0015986);psbF(GO:0009767);rps7(GO:0002181,GO:0006364);psbE(GO:0009767);petB(GO:0022904,GO:0015979);rpl16(GO:0006412);petG(GO:0017148,GO:0015979);petA(GO:0015979);rps12(GO:0006412);ndhH(GO:0019684);rpl2(GO:0006412);rps3(GO:0006412);rps2(GO:0006412);atpB(GO:0015986,GO:0015991);ndhE(GO:0019684,GO:0042773);psaA(GO:0018298,GO:0015979);psbT(GO:0015979);psbM(GO:0019684);infA(GO:0006446);rpoB(GO:0006144,GO:0006206,GO:0006351);rpl36(GO:0006412);psbC(GO:0009772,GO:0018298);ndhD(GO:0006120,GO:0006744);rpoC1(GO:0006144,GO:0006351,GO:0006206);atpE(GO:0006119,GO:0015986,GO:0015991);psbN(GO:0015979);rps11(GO:0006412,GO:0006144,GO:0006206,GO:0006351);rps4(GO:0006412);psaI(GO:0015979);ndhC(GO:0006120,GO:0019684,GO:0006744);psaJ(GO:0015979);rps18(GO:0006412);psbL(GO:0009767);rpl33(GO:0006412);ndhA(GO:0019684);rpl23(GO:0006412);rps19(GO:0006412);ndhG(GO:0006120,GO:0006744);rpl32(GO:0006412);ndhK(GO:0006120,GO:0019684,GO:0006744);rpl14(GO:0006412);psaC(GO:0009773);psbJ(GO:0015979);rpl22(GO:0006412);rps8(GO:0006412);ndhB(GO:0006120,GO:0019684,GO:0006744);psbH(GO:0015979);rpoC2(GO:0006144,GO:0006351,GO:0006206);rbcL(GO:0019253,GO:0046487,GO:0009853);ndhJ(GO:0006120,GO:0019684,GO:0006744);atpI(GO:0015986);psbB(GO:0009767,GO:0018298);rpoA(GO:0006144,GO:0006206,GO:0006351);ycf3(GO:0015979);psbD(GO:0009772);cemA(GO:1902600);rps16(GO:0006412);psbZ(GO:0042549);rps14(GO:0006412);petD(GO:0009767);atpA(GO:0015986,GO:0006119,GO:0015991);psaB(GO:0018298,GO:0015979);rps15(GO:0006412);psbI(GO:0015979);psbA(GO:0009772);ycf4(GO:0015979) |
| biological_process | response to stimulus | GO:0050896 | 2 | petG(GO:0009411,GO:0046688);psbA(GO:0009635) |
| biological_process | single-organism process | GO:0044699 | 38 | ndhC(GO:0006120,GO:0006744,GO:0006814,GO:0015992);psbL(GO:0009767);atpH(GO:0015986,GO:0015991);ndhI(GO:0006120,GO:0006744,GO:0006814,GO:0015992);ndhA(GO:0055114);ndhG(GO:0006120,GO:0006744,GO:0015992,GO:0006814);ndhK(GO:0006120,GO:0006744,GO:0006814,GO:0015992);ndhF(GO:0006120,GO:0006744,GO:0015992,GO:0006814);atpF(GO:0015986);psbF(GO:0009767);psaC(GO:0009773);psbE(GO:0009767);petB(GO:0022904);ndhB(GO:0006120,GO:0006744,GO:0006814,GO:0015992);petG(GO:0055114);petA(GO:0055114);ndhH(GO:0055114);rpoC2(GO:0006144,GO:0006206);rbcL(GO:0019253,GO:0046487,GO:0055114);ndhJ(GO:0006120,GO:0006744,GO:0015992,GO:0006814);atpI(GO:0015986);psbB(GO:0009767);rpoA(GO:0006144,GO:0006206);atpB(GO:0015986,GO:0015991);psbD(GO:0009772);ndhE(GO:0042773);cemA(GO:1902600);psaA(GO:0055114);rpoB(GO:0006144,GO:0006206);psbC(GO:0009772);petD(GO:0009767);ndhD(GO:0006120,GO:0006744,GO:0015992,GO:0006814);atpA(GO:0015986,GO:0006119,GO:0015991);rpoC1(GO:0006144,GO:0006206);atpE(GO:0006119,GO:0015986,GO:0015991);rps11(GO:0006144,GO:0006206);psaB(GO:0055114);psbA(GO:0009772) |
| biological_process | negative regulation of biological process | GO:0048519 | 1 | petG(GO:0017148) |
| biological_process | localization | GO:0051179 | 17 | ndhJ(GO:0015992,GO:0006814);ndhC(GO:0006814,GO:0015992);atpI(GO:0015986);atpH(GO:0015986,GO:0015991);ndhI(GO:0006814,GO:0015992);atpB(GO:0015986,GO:0015991);ndhG(GO:0015992,GO:0006814);ndhE(GO:0006810);cemA(GO:1902600);ndhK(GO:0006814,GO:0015992);ndhF(GO:0015992,GO:0006814);atpF(GO:0015986);ndhD(GO:0015992,GO:0006814);atpA(GO:0015986,GO:0015991);atpE(GO:0015986,GO:0015991);ndhB(GO:0006814,GO:0015992);ndhH(GO:0006810) |
| biological_process | metabolic process | GO:0008152 | 75 | atpH(GO:0015986);ndhI(GO:0006120,GO:0019684,GO:0006744);psbK(GO:0015979);rpl20(GO:0006412);matK(GO:0006397,GO:0008380,GO:0008033);ndhF(GO:0006120,GO:0006744);atpF(GO:0015986);psbF(GO:0009767);rps7(GO:0002181,GO:0006364);psbE(GO:0009767);petB(GO:0022904,GO:0015979);rpl16(GO:0006412);petG(GO:0017148,GO:0055114,GO:0015979);petA(GO:0055114,GO:0015979);rps12(GO:0006412);ndhH(GO:0019684,GO:0055114);rpl2(GO:0006412);clpP(GO:0006508);rps3(GO:0006412);rps2(GO:0006412);atpB(GO:0015986);ndhE(GO:0019684,GO:0042773);psaA(GO:0018298,GO:0055114,GO:0015979);psbT(GO:0015979);psbM(GO:0019684);infA(GO:0006446);rpoB(GO:0006144,GO:0006206,GO:0006351);rpl36(GO:0006412);psbC(GO:0009772,GO:0018298);ndhD(GO:0006120,GO:0006744);rpoC1(GO:0006144,GO:0006351,GO:0006206);atpE(GO:0006119,GO:0015986);psbN(GO:0015979);rps11(GO:0006412,GO:0006144,GO:0006206,GO:0006351);rps4(GO:0006412);psaI(GO:0015979);ndhC(GO:0006120,GO:0019684,GO:0006744);psaJ(GO:0015979);rps18(GO:0006412);psbL(GO:0009767);rpl33(GO:0006412);ndhA(GO:0019684,GO:0055114);rpl23(GO:0006412);rps19(GO:0006412);ndhG(GO:0006120,GO:0006744);rpl32(GO:0006412);ndhK(GO:0006120,GO:0019684,GO:0006744);rpl14(GO:0006412);psaC(GO:0009773);psbJ(GO:0015979);rpl22(GO:0006412);rps8(GO:0006412);ndhB(GO:0006120,GO:0019684,GO:0006744);psbH(GO:0050821,GO:0015979);rpoC2(GO:0006144,GO:0006351,GO:0006206);rbcL(GO:0019253,GO:0046487,GO:0009853,GO:0055114);ndhJ(GO:0006120,GO:0019684,GO:0006744);atpI(GO:0015986);psbB(GO:0009767,GO:0018298);rpoA(GO:0006144,GO:0006206,GO:0006351);ycf3(GO:0015979);psbD(GO:0009772);rps16(GO:0006412);psbZ(GO:0042549);rps14(GO:0006412);petD(GO:0009767);atpA(GO:0015986,GO:0006119);psaB(GO:0018298,GO:0055114,GO:0015979);rps15(GO:0006412);psbI(GO:0015979);psbA(GO:0009772);ycf4(GO:0015979);petN(GO:0017004,GO:0015979);petL(GO:0015979); |
| biological_process | biological regulation | GO:0065007 | 4 | infA(GO:0006446);petG(GO:0055070,GO:0017148);psbH(GO:0050821);psbZ(GO:0042549) |
