# Supplementary material for: The complete chloroplast genomes of seventeen Aegilops tauschii: genome comparative analysis and phylogenetic inference
Source: PeerJ. 2020 Mar 4;8:e8678. doi: 10.7717/peerj.8678 (PMC7060751; doi:10.7717/peerj.8678)
Supplement: Table S3 [file peerj-08-8678-s004.docx]

**Table S2**

| location | Detailed location | Regions | Nucleotide diversity（Pi） |
| --- | --- | --- | --- |
| LSC | Noncoding | atpA-trnR-UCU | 0.00087 |
| LSC | Noncoding | cemA-coding | 0.00051 |
| LSC | Noncoding | petA-psbJ | 0.00054 |
| LSC | Noncoding | petD-rpoA | 0.0027 |
| LSC | psbK-psbI | psbK-psbI | 0.00192 |
| LSC | psbM-petN | psbM-petN | 0.00035 |
| LSC | rps2-atpI | rps2-atpI | 0.00043 |
| LSC | rps11-rpl36 | rps11-rpl36 | 0.00285 |
| LSC | rps14-psaB | rps14-psaB | 0.00302 |
| LSC | rps16 intron | rps16 intron | 0.00015 |
| LSC | rps16-trnQ-UUG | rps16-trnQ-UUG | 0.00128 |
| LSC | rps18-rpl20 | rps18-rpl20 | 0.00635 |
| LSC | trnD-GUC-psbM | trnD-GUC-psbM | 0.00029 |
| LSC | trnP-UGG-psaJ | trnP-UGG-psaJ | 0.00275 |
| LSC | trnQ-UUG-psbK | trnQ-UUG-psbK | 0.00128 |
| LSC | ycf4-cemA | ycf4-cemA | 0.00161 |
| LSC | atpB-rbcL | atpB-rbcL | 0.00015 |
| LSC | atpH-atpF | atpH-atpF | 0.0012 |
| LSC | ndhC-trnM-CAU | ndhC-trnM-CAU | 0.0002 |
| LSC | petN-trnC-GCA | petN-trnC-GCA | 0.00012 |
| LSC | psbA-matK | psbA-matK | 0.00022 |
| LSC | psbE-petL | psbE-petL | 0.00087 |
| LSC | psbH-petB | psbH-petB | 0.00028 |
| LSC | rbcL-psaI | rbcL-psaI | 0.00492 |
| LSC | rpl16-rps3 | rpl16-rps3 | 0.00092 |
| LSC | tRNAK intrion | tRNAK intrion | 0.00027 |
| LSC | tRNAK-rps16 | tRNAK-rps16 | 0.00067 |
| LSC | trnC-GCA-rpoB | trnC-GCA-rpoB | 0.00221 |
| LSC | trnF-GAA-ndhJ | trnF-GAA-ndhJ | 0.00055 |
| LSC | trnfM-CAU-trnT-GGU | trnfM-CAU-trnT-GGU | 0.00012 |
| LSC | trnS-GCU-psbD | trnS-GCU-psbD | 0.00023 |
| LSC | trnT-UGU-trnF-GAA | trnT-UGU-trnF-GAA | 0.00069 |
| LSC | trnY-GUA-trnD-GUC | trnY-GUA-trnD-GUC | 0.00067 |
| LSC | ycf3-intron | ycf3-intron | 0.00008 |
| SSC | ndhA-intron | ndhA-intron | 0.00051 |
| SSC | ndhG-ndhI | ndhG-ndhI | 0.00046 |
| SSC | ccsA-ndhD | ccsA-ndhD | 0.00483 |
| SSC | ndhF-rpl32 | ndhF-rpl32 | 0.00013 |
| SSC | rpl32-trnL-UAG | rpl32-trnL-UAG | 0.00478 |
