# Supplementary material for: The complete chloroplast genomes of seventeen Aegilops tauschii: genome comparative analysis and phylogenetic inference
Source: PeerJ. 2020 Mar 4;8:e8678. doi: 10.7717/peerj.8678 (PMC7060751; doi:10.7717/peerj.8678)
Supplement: Table S4 [file peerj-08-8678-s005.docx]

**Table S3**

| Region | Pairs | Primers | Product size (bp) |
| --- | --- | --- | --- |
| *rbcL*-psaI | F1 | GATAAAACTAAAGATAAAGAAGGTA | 619 |
|  | R1 | ATAGGTGTCTCAATTCAAATTTACT |  |
| *rps18*-rpl20 | F2 | ATAAAGAAAGTAATCCAGCTCGTTG | 105 |
|  | R2 | TATACTGGAATAATAAAAGATGAAT |  |
| *rpl32*-trnL-UAG | F3 | TTCTTCTTTGAATGTACTTTTATGT | 662 |
|  | R3 | TCAAATAATAGGTAACTTAAAAGAA |  |
| *ccsA*-ndhD | F4 | CTAAATGATTACATAAAATAAAACC | 145 |
|  | R4 | TAATGCAGAAATAAGTAAAAAAGTA |  |
