# Supplementary figures and images for: The complete chloroplast genomes of seventeen Aegilops tauschii: genome comparative analysis and phylogenetic inference
Source: PeerJ. 2020 Mar 4;8:e8678. doi: 10.7717/peerj.8678 (PMC7060751; doi:10.7717/peerj.8678)

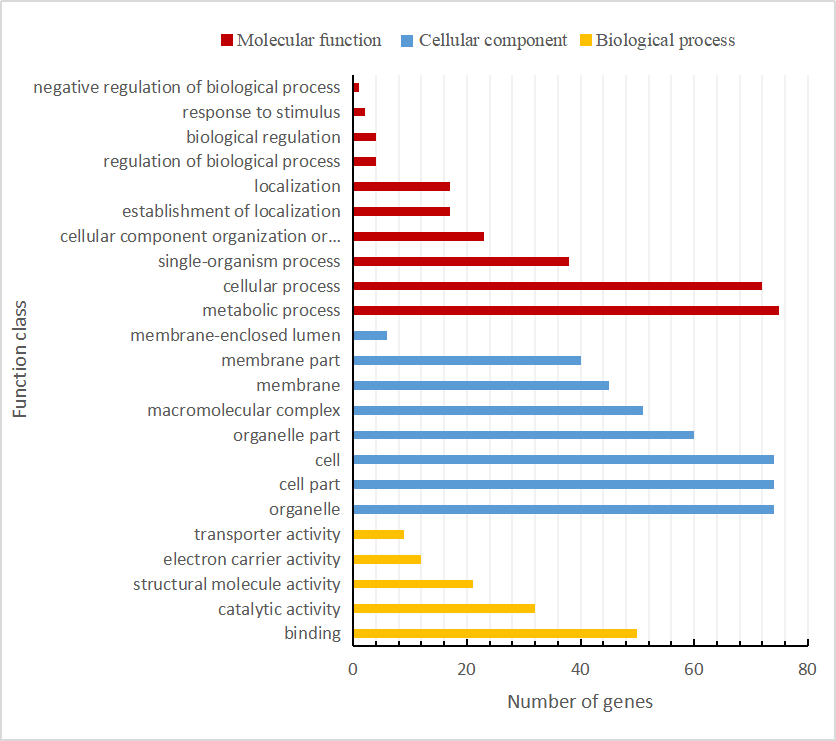

Supplement: Figure S1 [file peerj-08-8678-s006.png]

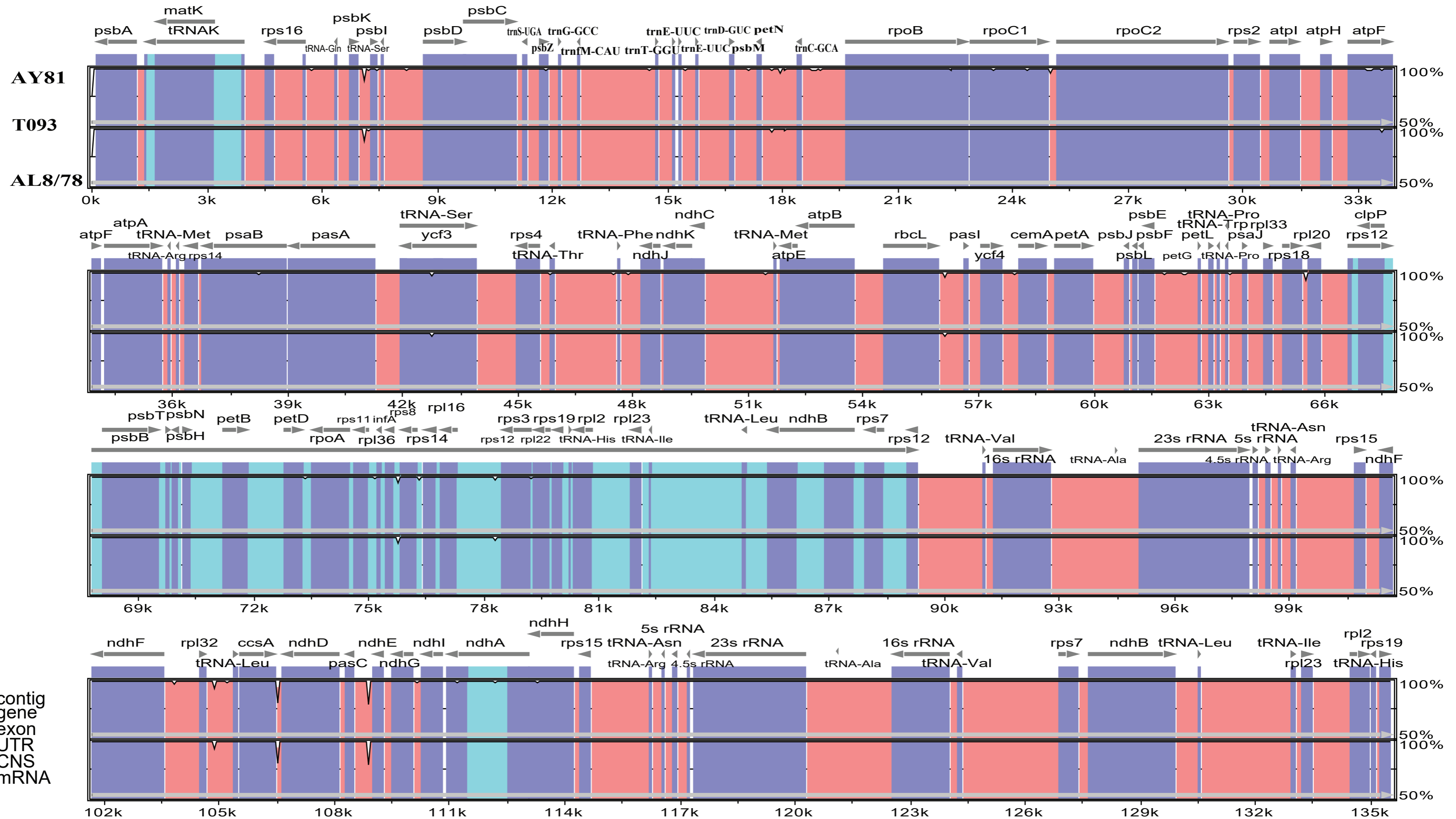

Supplement: Figure S2 — The vertical scale of mVISTA visualization image denotes the percentage of identity, ranging from 50% to 100%. The horizontal axis marked color code as protein-coding genes, rRNA, tRNA or conserved non-coding regions. [file peerj-08-8678-s007.pdf]

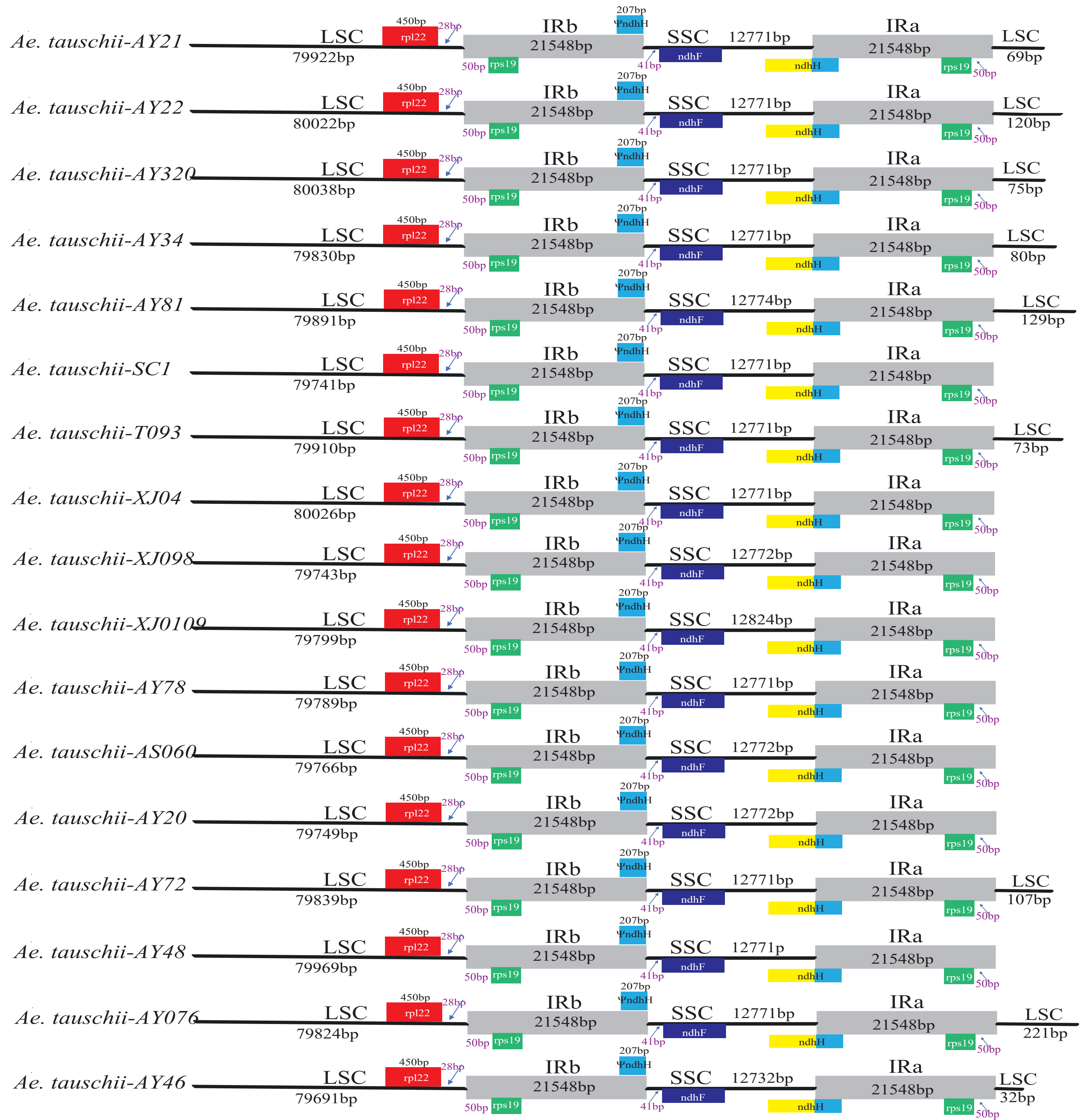

Supplement: Fgirue S3 [file peerj-08-8678-s008.pdf]
